# Supplementary material for: Whole genome case-control study of central nervous system toxicity due to antimicrobial drugs
Source: PLoS One. 2024 Feb 29;19(2):e0299075. doi: 10.1371/journal.pone.0299075 (PMC10903854; doi:10.1371/journal.pone.0299075)
Supplement: S1 Fig — (DOCX) [file pone.0299075.s001.docx]

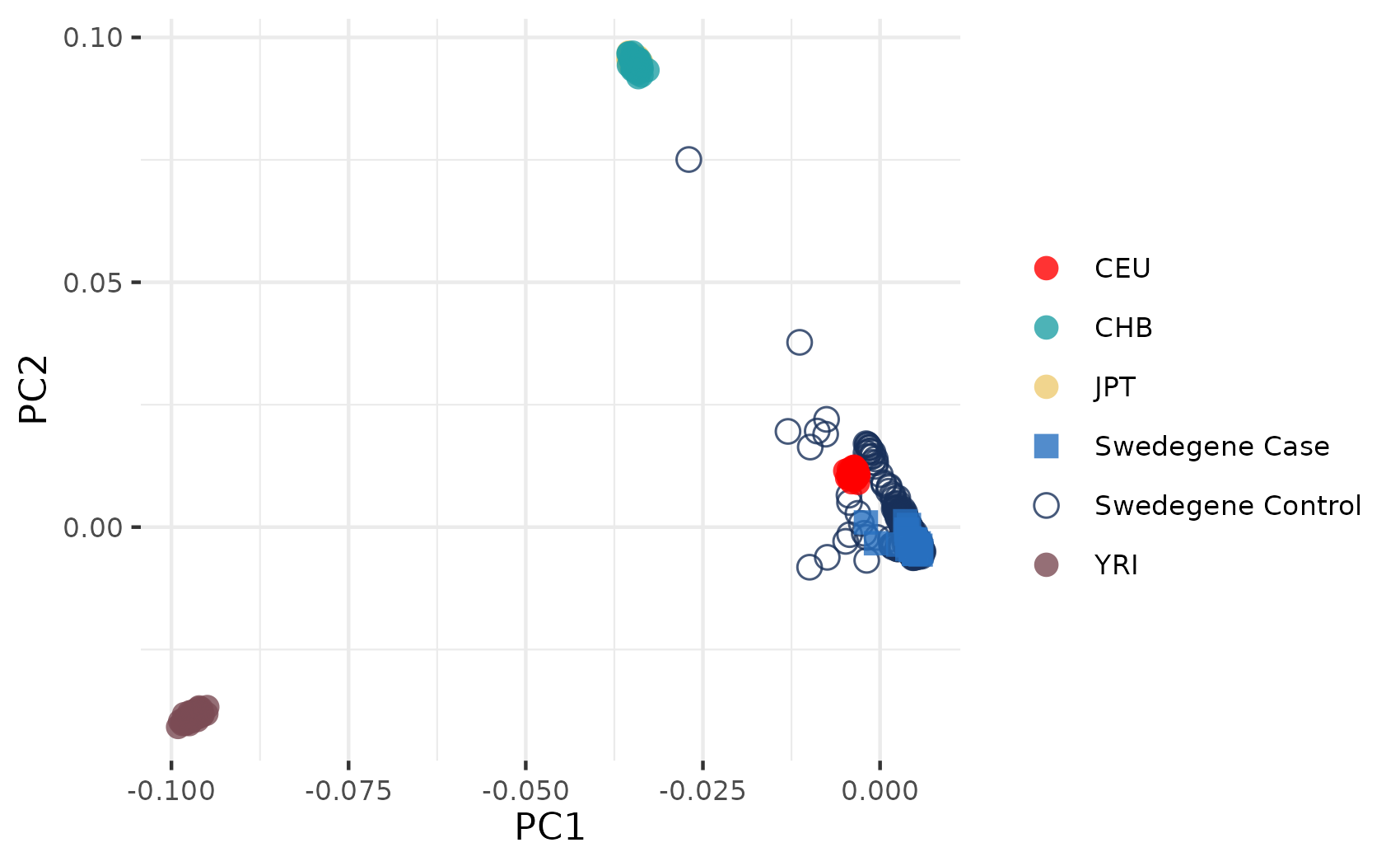
**Figure S1**. Principal component analysis plot of the Swedegene cohort merged with HapMap r23a. Swedegene controls are depicted as dark blue circles and cases as light blue squares. Number of individuals in each group, CEU: 90, CHB: 45, JBT:45, Swedegene controls: 833, Swedegene cases: 66, YRI:90
