# Supplementary material for: Whole genome case-control study of central nervous system toxicity due to antimicrobial drugs
Source: PLoS One. 2024 Feb 29;19(2):e0299075. doi: 10.1371/journal.pone.0299075 (PMC10903854; doi:10.1371/journal.pone.0299075)
Supplement: S2 Fig — (DOCX) [file pone.0299075.s002.docx]

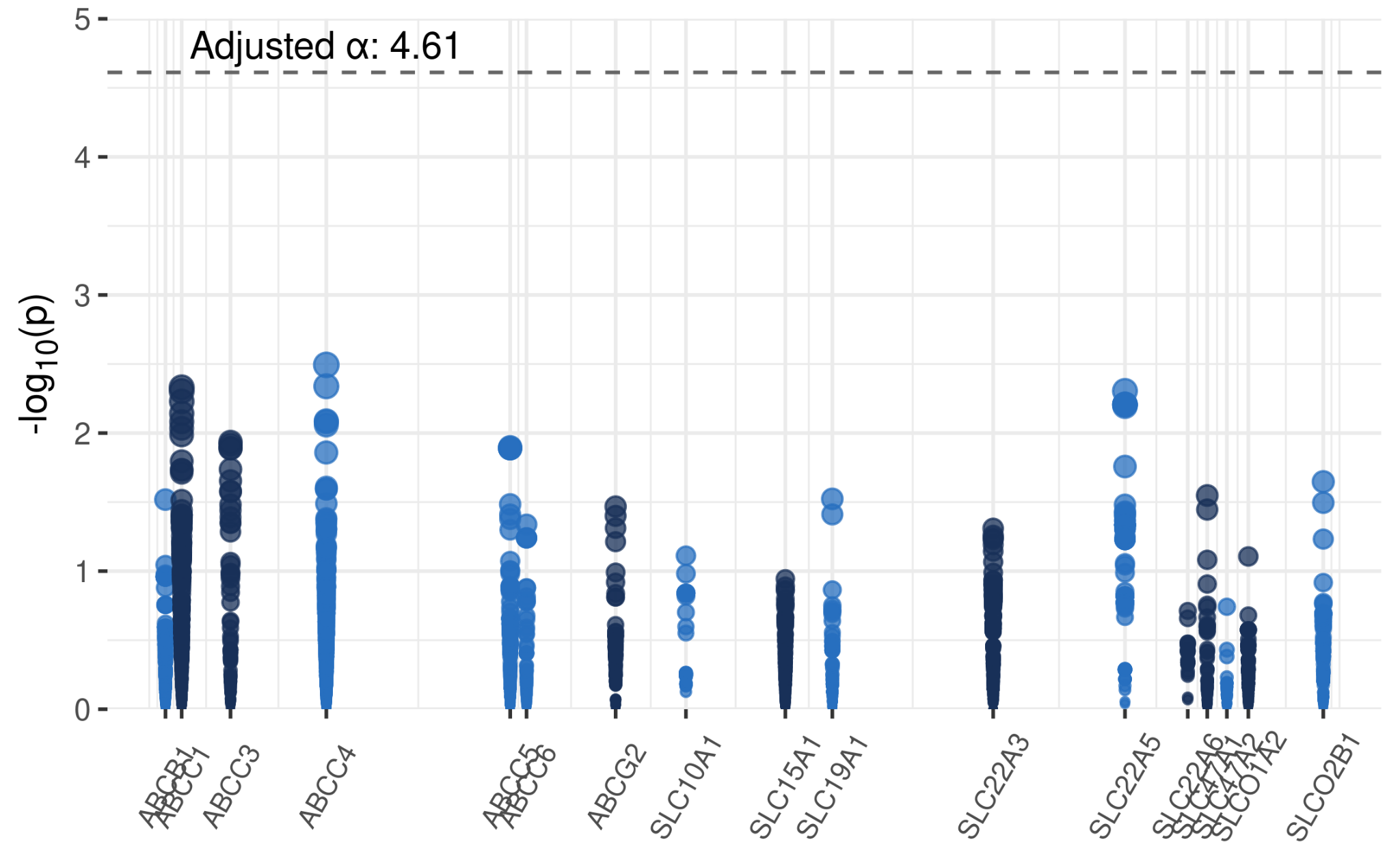


**Figure S2**. Association p values of candidate gene variants with a variant frequency of at least 0.123 in cases with CNS toxicity (n=66) vs controls (n=833). Association was tested with logistic regression with the firth fallback option in PLINK2.0, and principal components one to four as covariates. The significance threshold p < 2.45 x 10^-5^ (e-4.61, dotted line) was calculated using Bonferroni correction.
