# Supplementary material for: Whole genome case-control study of central nervous system toxicity due to antimicrobial drugs
Source: PLoS One. 2024 Feb 29;19(2):e0299075. doi: 10.1371/journal.pone.0299075 (PMC10903854; doi:10.1371/journal.pone.0299075)
Supplement: S3 Fig — (DOCX) [file pone.0299075.s003.docx]

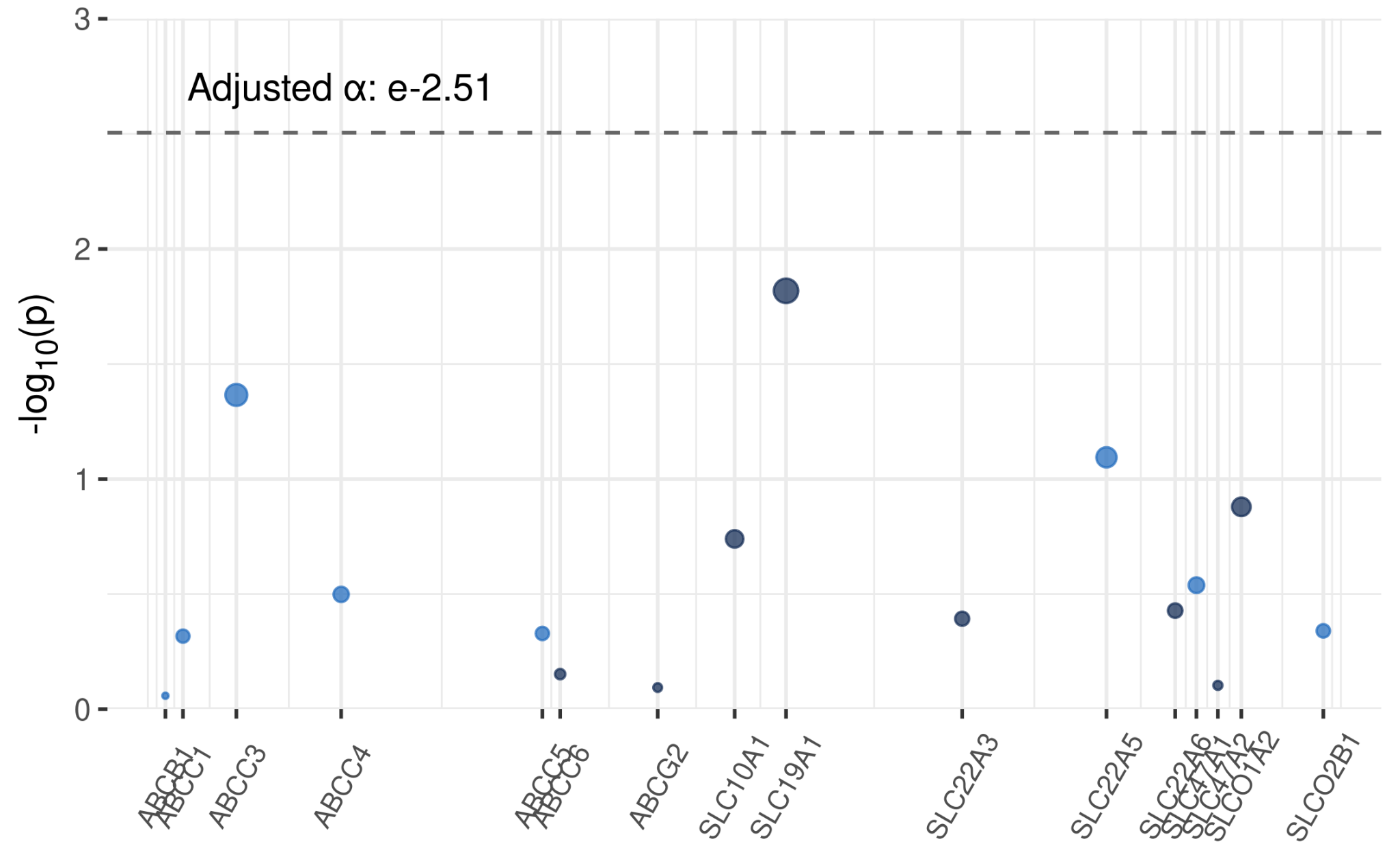


**Figure S3**. Association p values of SKAT-O gene tests in cases with CNS toxicity (n=66) vs controls (n=833). All variants included in each test are closer than 140 bp to an exon, 3’ UTR or 5’-UTR of the gene. Additionally, each selected variant has a variant frequency below 0.123. Principal components one to four were added as covariates and significance threshold p < 3.09 x 10^-3^ (e-2.51, dotted line) was calculated using Bonferroni correction.
