# Supplementary material for: Whole genome case-control study of central nervous system toxicity due to antimicrobial drugs
Source: PLoS One. 2024 Feb 29;19(2):e0299075. doi: 10.1371/journal.pone.0299075 (PMC10903854; doi:10.1371/journal.pone.0299075)
Supplement: S4 Fig — (DOCX) [file pone.0299075.s004.docx]

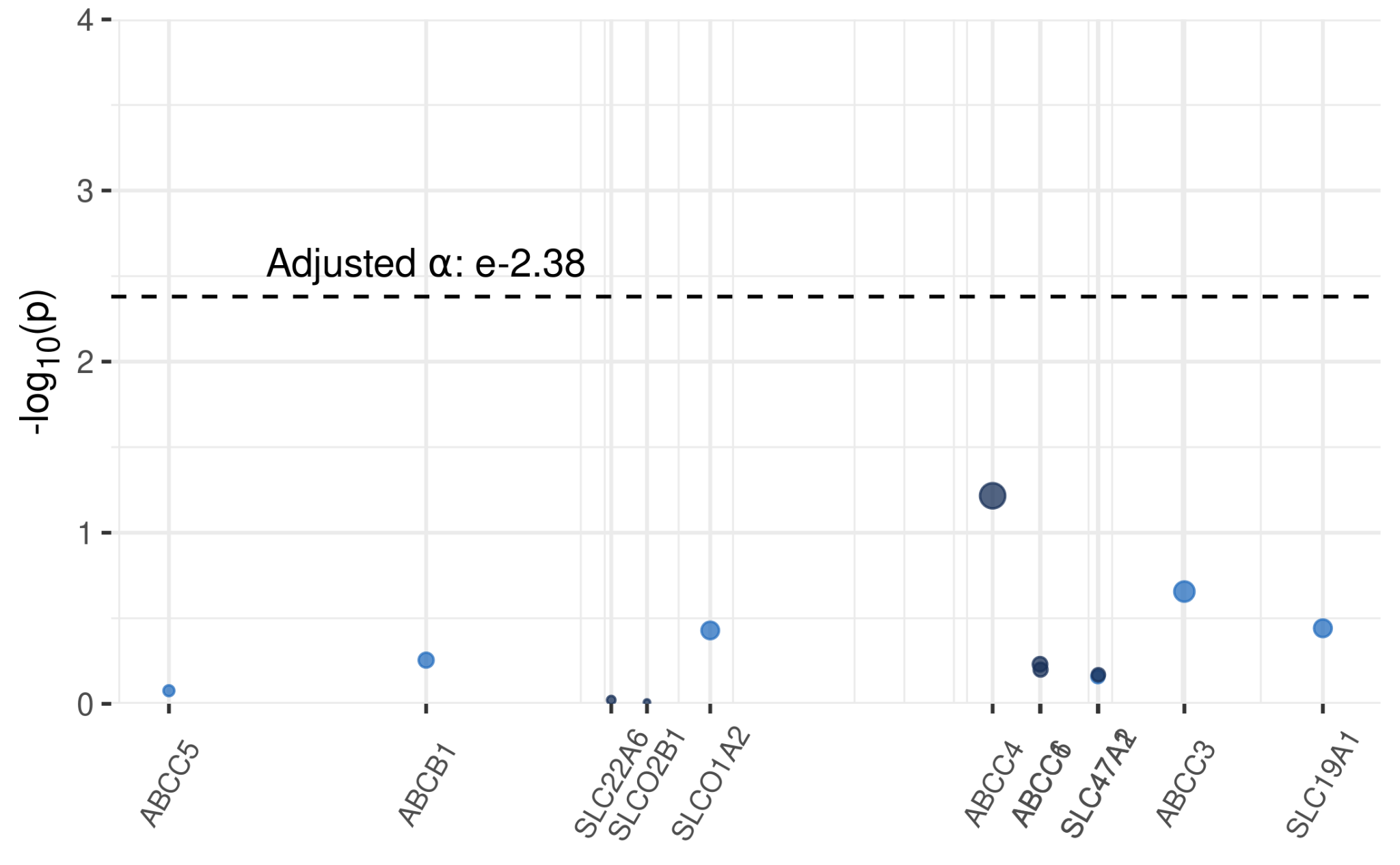


**Figure S4**. Association p values of structural variation in a dominant model in cases with CNS toxicity (n=66) vs controls (n=833). Association was tested with logistic regression. The significance threshold p < 4.17 x 10^-3^ (e-2.38, dotted line) was calculated using Bonferroni correction.
