# Supplementary material for: Whole genome case-control study of central nervous system toxicity due to antimicrobial drugs
Source: PLoS One. 2024 Feb 29;19(2):e0299075. doi: 10.1371/journal.pone.0299075 (PMC10903854; doi:10.1371/journal.pone.0299075)
Supplement: S5 Fig — (DOCX) [file pone.0299075.s005.docx]

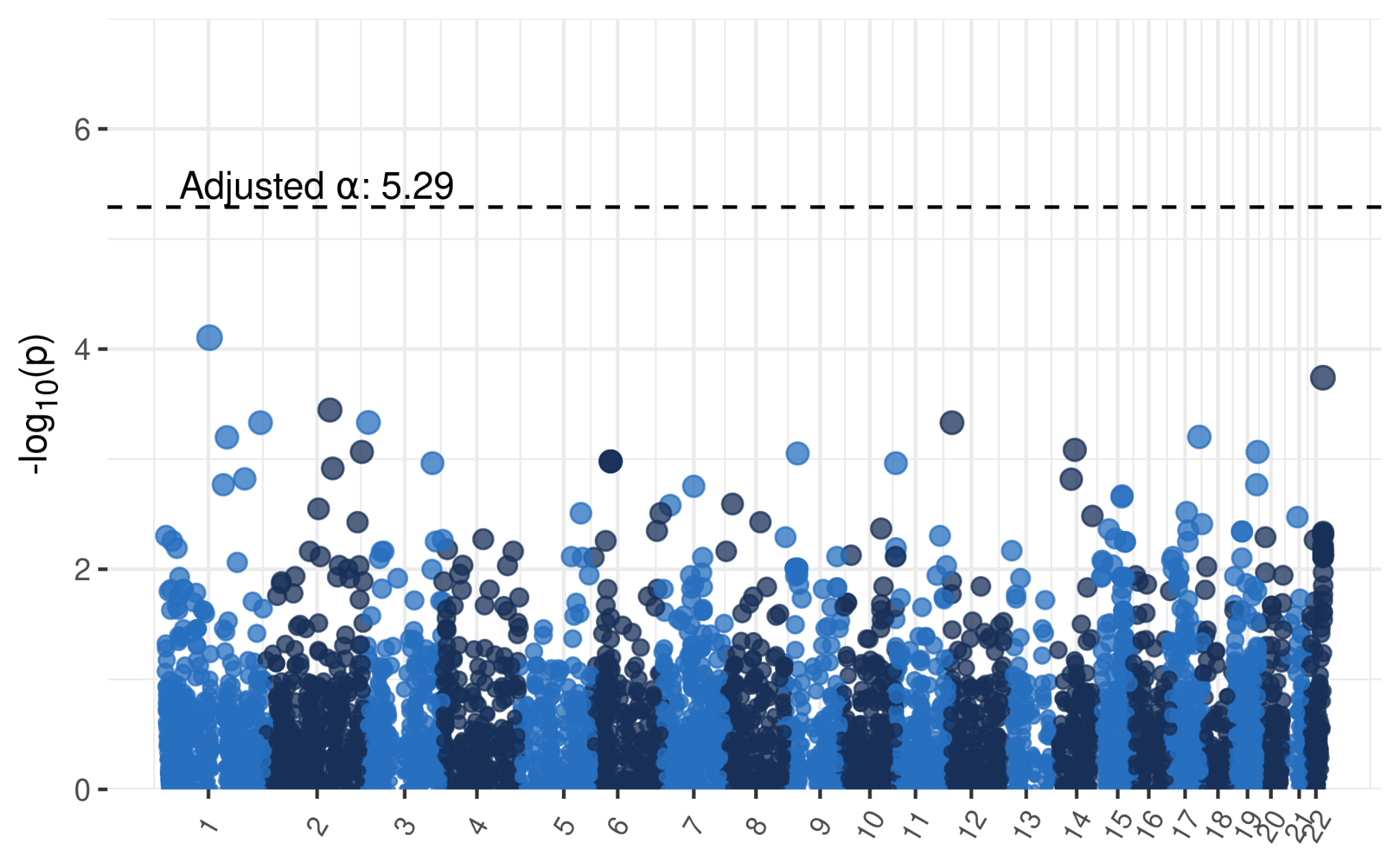
**Figure S5.** Dominant test for structural variation in genes in cases with CNS toxicity (n=66) vs controls (n=833). The significance threshold p < 5.12 x 10^-6^ (e-5.29, dotted line) was adjusted using Bonferroni correction.
