# Supplementary material for: Whole genome case-control study of central nervous system toxicity due to antimicrobial drugs
Source: PLoS One. 2024 Feb 29;19(2):e0299075. doi: 10.1371/journal.pone.0299075 (PMC10903854; doi:10.1371/journal.pone.0299075)
Supplement: S6 Fig — (DOCX) [file pone.0299075.s006.docx]

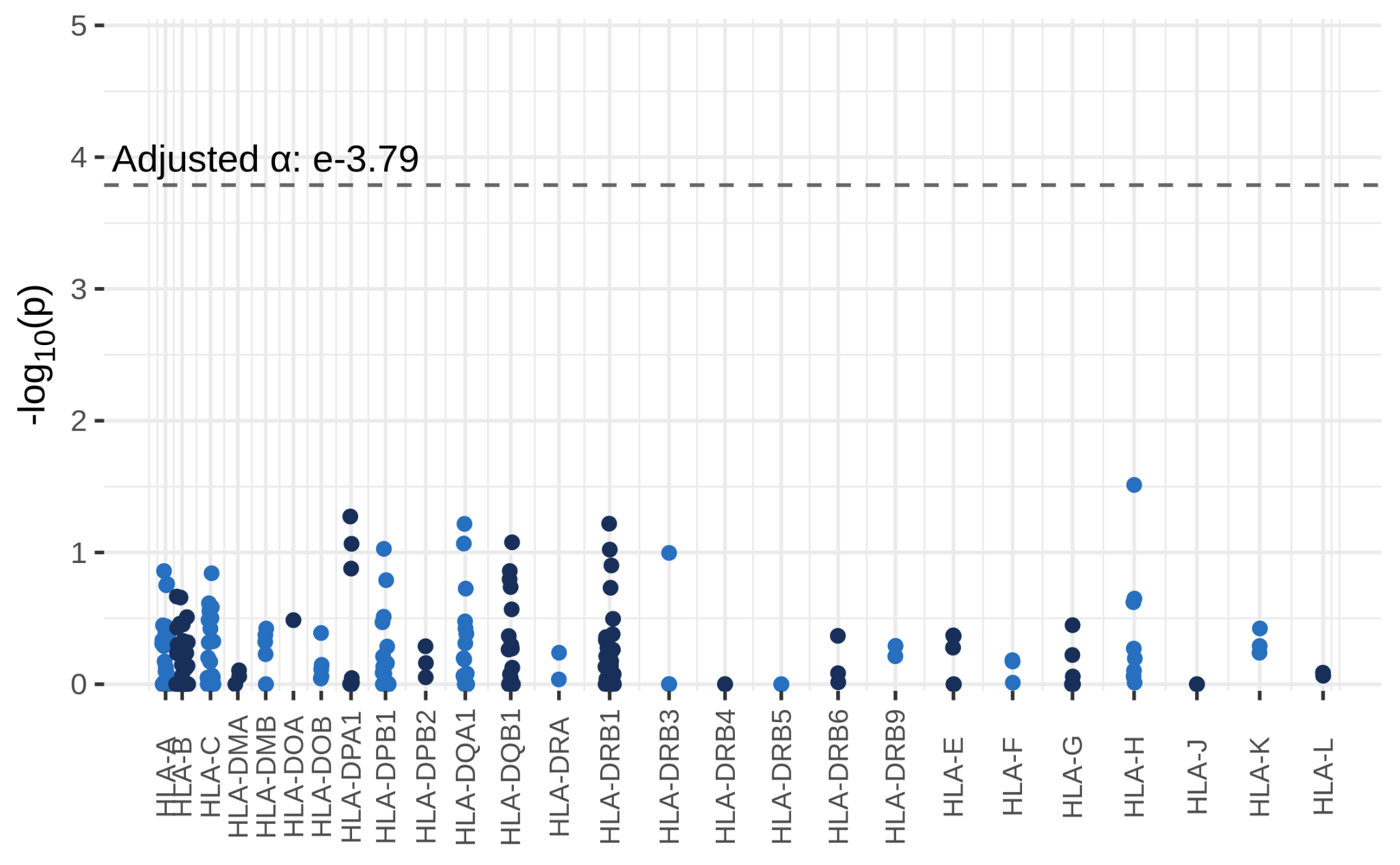


**Figure S6:** Four-digit HLA type association test with a dominant model, grouped on locus, in cases with CNS toxicity (n=66) vs controls (n=833). The significance threshold p < 1.62 x 10^-4^ (e-3.79, dotted line) was calculated using Bonferroni correction.
