# Supplementary material for: Whole genome case-control study of central nervous system toxicity due to antimicrobial drugs
Source: PLoS One. 2024 Feb 29;19(2):e0299075. doi: 10.1371/journal.pone.0299075 (PMC10903854; doi:10.1371/journal.pone.0299075)
Supplement: S1 Table — (DOCX) [file pone.0299075.s007.docx]

**Table S1.** Published data on the role of drug transporters for the suspected drugs.

| **Drug** | **Gene** | **Type of experimental model** | **Outcomes** | **Major Findings** | **References** (1st author, year) | **Notes** |
| --- | --- | --- | --- | --- | --- | --- |
| Aciclovir | *ABCB1* (MDR-1) | In vitro | PK | Substrate for P-gp | Palmberger 2008 [[1]](https://paperpile.com/c/5sDUfm/jvJLy) |  |
| Amoxicillin |  |  |  |  |  | Unknown, not studied |
| Atovaquone | Not substrate for P-gp, OATP1B1, OATP1B3, OATP2B1, OCT1, NTCP, OAT2, BCRP |  |  |  | Patel 2018 [[2]](https://paperpile.com/c/5sDUfm/q7a1l) |  |
| Azithromycin | *ABCB1* (MDR-1) | Clinical | PK | 2677TT/3435TT associated with lower Cmax/higher Tmax | He X.J 2009 [[3]](https://paperpile.com/c/5sDUfm/wuCBc) |  |
| Azithromycin | *ABCB1* (MDR-1) | In vitro | PK | Substrate for P-gp | Horita 2014 [[4]](https://paperpile.com/c/5sDUfm/Bs697) |  |
| Ceftibuten | *SLC15A1* (PEPT1) | In vitro | PK | Substrate for PEPT1 | Irie 2005 [[5]](https://paperpile.com/c/5sDUfm/uewmd) |  |
| Chloroquine |  |  |  |  |  | Unknown, not studied |
| Ciprofloxacin | *ABCG2* (BCRP) | In vitro | PK | Substrate for BCRP | Haslam 2011 [[6]](https://paperpile.com/c/5sDUfm/V9oV7) |  |
| Ciprofloxacin | *ABCG2* (BCRP) | In vitro | PK | Substrate for BCRP | Merino 2006 [[7]](https://paperpile.com/c/5sDUfm/ryZbZ) |  |
| Clarithromycin | *ABCB1* (MDR-1) | In vitro | PK | Substrate for P-gp | Pachot 2003 [[8]](https://paperpile.com/c/5sDUfm/FrzHc) |  |
| Clarithromycin | *ABCB1* (MDR-1) | In vitro | PK | Substrate for P-gp | Horita 2014 [[4]](https://paperpile.com/c/5sDUfm/Bs697) |  |
| Clavulanic acid |  |  |  |  |  | Unknown, not studied |
| Doxycycline | *ABCB1* (MDR-1) | In vitro | PK | Substrate for P-gp | Mealey 2002 [[9]](https://paperpile.com/c/5sDUfm/qkAu8) |  |
| Etoricoxib |  |  |  |  |  | Unknown, not studied |
| Gentamycin |  |  |  |  |  | Unknown, not studied |
| Hydroxychloroquine |  |  |  |  |  | Unknown, not studied |
| Immunoglobulin |  |  |  |  |  | Unknown, not studied |
| Lamivudine | *ABCB1* (MDR-1) | Clinical |  | rs1045642  (3435C>T) associated with increased  resistance  to lamivudine | Coelho Antonio 2013 [[10]](https://paperpile.com/c/5sDUfm/XBiRe) |  |
| Lamivudine | *ABCC1* | Clinical |  | rs212091 198217T>C;  3'-UTR associated with increased  resistance  to lamivudine | Coelho Antonio 2014 [[11]](https://paperpile.com/c/5sDUfm/ZLL7x) |  |
| Lamivudine | *ABCB1* (MDR-1) | In vitro |  | Substrate for P-gp | de Souza 2009 [[12]](https://paperpile.com/c/5sDUfm/XXMkb) |  |
| Levofloxacin | *SLCO1A2* (OATP1A2) | In vitro | PK | Substrate for OATP1A2 | Maeda 2007 [[13]](https://paperpile.com/c/5sDUfm/dydY7) |  |
| Levofloxacin | *ABCB1* (MDR-1) | In vitro | PK | Substrate for P-gp | Ito 1997 [[14]](https://paperpile.com/c/5sDUfm/d38nh) |  |
| Linezolid | *ABCB1* (MDR-1) | Clinical | PK | rs1045642  associated with lower clearance | Allegra 2018 [[15]](https://paperpile.com/c/5sDUfm/uEntf) |  |
| Linezolid | *ABCB1* (MDR-1) | Clinical | PK | Clarithomycin increased concentration of linezolide | Bolhuis 2013 [[16]](https://paperpile.com/c/5sDUfm/fj1RW) |  |
| Linezolid | *ABCB1* (MDR-1) | Clinical | PK | P-gp inhibitors increased linezolide exposure | Pea 2010 [[17]](https://paperpile.com/c/5sDUfm/7at9v) |  |
| Lymecycline |  |  |  |  |  | Unknown, not studied |
| Mefloquine | *ABCB1* (MDR-1) | Clinical | ADR risk, PK | 1236TT, 2677TT,  3435TT  associated with  neuropsychiatric ADRs in women | Aarnoudse 2006 [[18]](https://paperpile.com/c/5sDUfm/DVh8q) |  |
| Mefloquine | *ABCB1* (MDR-1) | case report | ADR risk | 3435TT, 2677TT associated with psychosis | Zaigraykina 2010 [[19]](https://paperpile.com/c/5sDUfm/S9TrH) |  |
| Metronidazole | *ABCB1* (MDR-1) | Clinical | PK | Possibly substrate for P-gp | Rajnarayana 2004 [[20]](https://paperpile.com/c/5sDUfm/OJwLQ) |  |
| Moxifloxacin | *ABCB1* (MDR-1) | Clinical | PK | rs2032582  (G2677T) associated with higher clearance | Naidoo 2018 [[21]](https://paperpile.com/c/5sDUfm/YKZgv) |  |
| Moxifloxacin | *ABCB1* (MDR-1) | Clinical | PK | rs1045642  (C3435T) associated with higher Tmax | Weiner 2007 [[22]](https://paperpile.com/c/5sDUfm/jDFWN) |  |
| Moxifloxacin | *ABCB1* (MDR-1) | In vitro | PK | Transported by P-gp | Brillault 2009 [[23]](https://paperpile.com/c/5sDUfm/ztdd8) |  |
| Moxifloxacin | *SLCO1B1* | Clinical | PK | rs4149015 associated with higher exposure and Cmax | Weiner M 2009 [[24]](https://paperpile.com/c/5sDUfm/kihe2) |  |
| Nitrofurantoin | *ABCG2* (BCRP) | In vitro | PK | Substrate for BCRP | Feinshtein 2010 [[25]](https://paperpile.com/c/5sDUfm/kVEjU) |  |
| Norfloxacin | *ABCG2* (BCRP) | In vitro | PK | Substrate for BCRP | Merino 2006 [[7]](https://paperpile.com/c/5sDUfm/ryZbZ) |  |
| Peginterferon alfa-2a |  |  |  |  |  | Unknown, not studied |
| Proguanil |  |  |  |  |  | Unknown, not studied |
| Ribavirin |  |  |  |  |  | Unknown, not studied |
| Sulfamethoxazole | Not substrate for P-gp, others unknown | In vitro | PK |  | Susanto 2002 [[26]](https://paperpile.com/c/5sDUfm/N55xy) |  |
| Terbinafine | Unknown, not studied |  |  |  |  |  |
| Tetracycline | *ABCB1* (MDR-1) | In vitro | PK | Substrate for P-gp | Kavallaris 1993 [[27]](https://paperpile.com/c/5sDUfm/YL702) |  |
| Tobramycin | *ABCB1* (MDR-1) | Studies in mice | PK | Substrate for P-gp | Banerjee 2000 [[28]](https://paperpile.com/c/5sDUfm/nBSxP) |  |
| Trimethoprim | *ABCB1* (MDR-1) | In vitro | PK | Substrate for P-gp | Susanto 2002 [[26]](https://paperpile.com/c/5sDUfm/N55xy) |  |
| Valaciclovir | *ABCB1* (MDR-1) | In vitro | PK | Substrate for P-gp | Palmberger 2008 [[1]](https://paperpile.com/c/5sDUfm/jvJLy) | Studied aciclovir |
| Vancomycin |  |  |  |  |  | Unknown, not studied |
| Varenicline |  |  |  |  |  | Unknown, not studied |
| Elvitegravir | *ABCB1* (MDR-1) | In vitro | PK | Substrate for P-gp | Gong 2020 [[29]](https://paperpile.com/c/5sDUfm/DR3ru) |  |
| Emtricitabine | MATE1 (not OCT1, OCT2, P-gp, BCRP or MRP2) | In vitro | PK | Substrate for MATE1 | Reznicek 2017 [[30]](https://paperpile.com/c/5sDUfm/uaL4Z) |  |
| Cobicistat |  |  |  |  |  | Unknown, not studied. Cobicistat is a P-gp inhibitor used to boost absorption. |
| Tenofovir | *ABCB1* (MDR-1) | In vitro | PK | Substrate for P-gp | Lepist 2012 [[31]](https://paperpile.com/c/5sDUfm/2iS9p) |  |
| Tenofovir | BCRP |  |  | Substrate for BCRP | SPC 2021 [[32]](https://paperpile.com/c/5sDUfm/EdWHX) |  |
| Dolutegravir | *ABCB1* (MDR-1) and BCRP | In vitro | PK | Substrate for P-gp and BCRP | Reese 2013 [[33]](https://paperpile.com/c/5sDUfm/0wIFA) |  |
| Dolutegravir | ABCB1 (MDR-1) and BCRP |  |  | Substrate for P-gp and BCRP | SPC 2020 [[34]](https://paperpile.com/c/5sDUfm/9AS4) |  |
| Lamivudine | *ABCB1* (MDR-1) | In vitro | PK | Substrate for P-gp | de Souza 2009 [[34]](https://paperpile.com/c/5sDUfm/9AS4) |  |
| Lamivudine | *ABCB1* (MDR-1) |  |  | Substrate for P-gp | SPC 2020 [[34]](https://paperpile.com/c/5sDUfm/9AS4) |  |
| Lamivudine | "OCT2" + MATE1 + MATE2 + BCRP + P-gp |  |  | Substrate for OCT2 + MATE1 + MATE2 + P-gp + BCRP | SPC 2020 [[34]](https://paperpile.com/c/5sDUfm/9AS4) |  |
| Phenoxymethylpenicillin |  |  |  |  |  | Unknown, not studied |

ADR = adverse drug reaction, PK = pharmacokinetic, SPC = summary of product characteristics.

**References**:

1. [Palmberger TF, Hombach J, Bernkop-Schnurch A. Thiolated chitosan: development and in vitro evaluation of an oral delivery system for acyclovir. Int J Pharm. 2008;348: 54–60.](http://paperpile.com/b/5sDUfm/jvJLy)

2. [Patel M, Johnson M, Sychterz CJ, Lewis GJ, Watson C, Ellens H, et al. Hepatobiliary Disposition of Atovaquone: A Case of Mechanistically Unusual Biliary Clearance. J Pharmacol Exp Ther. 2018;366: 37–45.](http://paperpile.com/b/5sDUfm/q7a1l)

3. [He XJ, Zhao LM, Qiu F, Sun YX, Li-Ling J. Influence of ABCB1 gene polymorphisms on the pharmacokinetics of azithromycin among healthy Chinese Han ethnic subjects. Pharmacol Rep. 2009;61: 843–850.](http://paperpile.com/b/5sDUfm/wuCBc)

4. [Horita Y, Doi N. Comparative study of the effects of antituberculosis drugs and antiretroviral drugs on cytochrome P450 3A4 and P-glycoprotein. Antimicrob Agents Chemother. 2014;58: 3168–3176.](http://paperpile.com/b/5sDUfm/Bs697)

5. [Irie M, Terada T, Katsura T, Matsuoka S, Inui K. Computational modelling of H+-coupled peptide transport via human PEPT1. J Physiol. 2005;565: 429–439.](http://paperpile.com/b/5sDUfm/uewmd)

6. [Haslam IS, Wright JA, O’Reilly DA, Sherlock DJ, Coleman T, Simmons NL. Intestinal ciprofloxacin efflux: the role of breast cancer resistance protein (ABCG2). Drug Metab Dispos. 2011;39: 2321–2328.](http://paperpile.com/b/5sDUfm/V9oV7)

7. [Merino G, Alvarez AI, Pulido MM, Molina AJ, Schinkel AH, Prieto JG. Breast cancer resistance protein (BCRP/ABCG2) transports fluoroquinolone antibiotics and affects their oral availability, pharmacokinetics, and milk secretion. Drug Metab Dispos. 2006;34: 690–695.](http://paperpile.com/b/5sDUfm/ryZbZ)

8. [Pachot JI, Botham RP, Haegele KD, Hwang K. Experimental estimation of the role of P-Glycoprotein in the pharmacokinetic behaviour of telithromycin, a novel ketolide, in comparison with roxithromycin and other macrolides using the Caco-2 cell model. J Pharm Pharm Sci. 2003;6: 1–12.](http://paperpile.com/b/5sDUfm/FrzHc)

9. [Mealey KL, Barhoumi R, Burghardt RC, Safe S, Kochevar DT. Doxycycline induces expression of P glycoprotein in MCF-7 breast carcinoma cells. Antimicrob Agents Chemother. 2002;46: 755–761.](http://paperpile.com/b/5sDUfm/qkAu8)

10. [Coelho AV, Silva SP, de Alencar LC, Stocco G, Crovella S, Brandao LA, et al. ABCB1 and ABCC1 variants associated with virological failure of first-line protease inhibitors antiretroviral regimens in Northeast Brazil patients. J Clin Pharmacol. 2013;53: 1286–1293.](http://paperpile.com/b/5sDUfm/XBiRe)

11. [Lienard E, Bouhsira E, Jacquiet P, Warin S, Kaltsatos V, Franc M. Efficacy of dinotefuran, permethrin and pyriproxyfen combination spot-on on dogs against Phlebotomus perniciosus and Ctenocephalides canis. Parasitol Res. 2013;112: 3799–3805.](http://paperpile.com/b/5sDUfm/ZLL7x)

12. [de Souza J, Benet LZ, Huang Y, Storpirtis S. Comparison of bidirectional lamivudine and zidovudine transport using MDCK, MDCK-MDR1, and Caco-2 cell monolayers. J Pharm Sci. 2009;98: 4413–4419.](http://paperpile.com/b/5sDUfm/XXMkb)

13. [Maeda T, Takahashi K, Ohtsu N, Oguma T, Ohnishi T, Atsumi R, et al. Identification of influx transporter for the quinolone antibacterial agent levofloxacin. Mol Pharm. 2007;4: 85–94.](http://paperpile.com/b/5sDUfm/dydY7)

14. [Ito T, Yano I, Tanaka K, Inui KI. Transport of quinolone antibacterial drugs by human P-glycoprotein expressed in a kidney epithelial cell line, LLC-PK1. J Pharmacol Exp Ther. 1997;282: 955–960.](http://paperpile.com/b/5sDUfm/d38nh)

15. [Allegra S, Di Paolo A, Cusato J, Fatiguso G, Arrigoni E, Danesi R, et al. A Common mdr1 Gene Polymorphism is Associated With Changes in Linezolid Clearance. Ther Drug Monit. 2018;40: 602–609.](http://paperpile.com/b/5sDUfm/uEntf)

16. [Bolhuis MS, van Altena R, van Soolingen D, de Lange WC, Uges DR, van der Werf TS, et al. Clarithromycin increases linezolid exposure in multidrug-resistant tuberculosis patients. Eur Respir J. 2013;42: 1614–1621.](http://paperpile.com/b/5sDUfm/fj1RW)

17. [Pea F, Furlanut M, Cojutti P, Cristini F, Zamparini E, Franceschi L, et al. Therapeutic drug monitoring of linezolid: a retrospective monocentric analysis. Antimicrob Agents Chemother. 2010;54: 4605–4610.](http://paperpile.com/b/5sDUfm/7at9v)

18. [Aarnoudse AL, van Schaik RH, Dieleman J, Molokhia M, van Riemsdijk MM, Ligthelm RJ, et al. MDR1 gene polymorphisms are associated with neuropsychiatric adverse effects of mefloquine. Clin Pharmacol Ther. 2006;80: 367–374.](http://paperpile.com/b/5sDUfm/DVh8q)

19. [Zaigraykina N, Potasman I. [Polymorphism at the MDR1 locus as a cause of mefloquine-induced psychosis]. Harefuah. 2010;149: 583–4, 620, 619.](http://paperpile.com/b/5sDUfm/S9TrH)

20. [Rajnarayana K, Reddy MS, Vidyasagar J, Krishna DR. Study on the influence of silymarin pretreatment on metabolism and disposition of metronidazole. Arzneimittelforschung. 2004;54: 109–113.](http://paperpile.com/b/5sDUfm/OJwLQ)

21. [Naidoo A, Ramsuran V, Chirehwa M, Denti P, McIlleron H, Naidoo K, et al. Effect of genetic variation in UGT1A and ABCB1 on moxifloxacin pharmacokinetics in South African patients with tuberculosis. Pharmacogenomics. 2018;19: 17–29.](http://paperpile.com/b/5sDUfm/YKZgv)

22. [Weiner M, Burman W, Luo CC, Peloquin CA, Engle M, Goldberg S, et al. Effects of rifampin and multidrug resistance gene polymorphism on concentrations of moxifloxacin. Antimicrob Agents Chemother. 2007;51: 2861–2866.](http://paperpile.com/b/5sDUfm/jDFWN)

23. [Brillault J, De Castro WV, Harnois T, Kitzis A, Olivier JC, Couet W. P-glycoprotein-mediated transport of moxifloxacin in a Calu-3 lung epithelial cell model. Antimicrob Agents Chemother. 2009;53: 1457–1462.](http://paperpile.com/b/5sDUfm/ztdd8)

24. [Weiner M, Gelfond J, Johnson-Pais TL, Engle M, Peloquin CA, Johnson JL, et al. Elevated Plasma Moxifloxacin Concentrations and SLCO1B1 g.-11187G>A Polymorphism in Adults with Pulmonary Tuberculosis. Antimicrob Agents Chemother. 2018;62. doi:](http://paperpile.com/b/5sDUfm/kihe2)[10.1128/AAC.01802-17](http://dx.doi.org/10.1128/AAC.01802-17)

25. [Feinshtein V, Holcberg G, Amash A, Erez N, Rubin M, Sheiner E, et al. Nitrofurantoin transport by placental choriocarcinoma JAr cells: involvement of BCRP, OATP2B1 and other MDR transporters. Arch Gynecol Obstet. 2010;281: 1037–1044.](http://paperpile.com/b/5sDUfm/kVEjU)

26. [Susanto M, Benet LZ. Can the enhanced renal clearance of antibiotics in cystic fibrosis patients be explained by P-glycoprotein transport? Pharm Res. 2002;19: 457–462.](http://paperpile.com/b/5sDUfm/N55xy)

27. [Kavallaris M, Madafiglio J, Norris MD, Haber M. Resistance to tetracycline, a hydrophilic antibiotic, is mediated by P-glycoprotein in human multidrug-resistant cells. Biochem Biophys Res Commun. 1993;190: 79–85.](http://paperpile.com/b/5sDUfm/YL702)

28. [Banerjee SK, Jagannath C, Hunter RL, Dasgupta A. Bioavailability of tobramycin after oral delivery in FVB mice using CRL-1605 copolymer, an inhibitor of P-glycoprotein. Life Sci. 2000;67: 2011–2016.](http://paperpile.com/b/5sDUfm/nBSxP)

29. [Gong Y, Chowdhury P, Nagesh PKB, Rahman MA, Zhi K, Yallapu MM, et al. Novel elvitegravir nanoformulation for drug delivery across the blood-brain barrier to achieve HIV-1 suppression in the CNS macrophages. Sci Rep. 2020;10: 3835.](http://paperpile.com/b/5sDUfm/DR3ru)

30. [Reznicek J, Ceckova M, Cerveny L, Muller F, Staud F. Emtricitabine is a substrate of MATE1 but not of OCT1, OCT2, P-gp, BCRP or MRP2 transporters. Xenobiotica. 2017;47: 77–85.](http://paperpile.com/b/5sDUfm/uaL4Z)

31. [Lepist EI, Phan TK, Roy A, Tong L, Maclennan K, Murray B, et al. Cobicistat boosts the intestinal absorption of transport substrates, including HIV protease inhibitors and GS-7340, in vitro. Antimicrob Agents Chemother. 2012;56: 5409–5413.](http://paperpile.com/b/5sDUfm/2iS9p)

32. [European Medicinces Agency. Genvoya - Summary of Product Characteristics. 2021. Available:](http://paperpile.com/b/5sDUfm/EdWHX) <https://www.ema.europa.eu/en/documents/product-information/genvoya-epar-product-information_en.pdf>

33. [Reese MJ, Savina PM, Generaux GT, Tracey H, Humphreys JE, Kanaoka E, et al. In vitro investigations into the roles of drug transporters and metabolizing enzymes in the disposition and drug interactions of dolutegravir, a HIV integrase inhibitor. Drug Metab Dispos. 2013;41: 353–361.](http://paperpile.com/b/5sDUfm/0wIFA)

34. [European Medicinces Agency. Dovato - Summary of Product Characteristics. 2020. Available:](http://paperpile.com/b/5sDUfm/9AS4) <https://www.ema.europa.eu/en/documents/product-information/dovato-epar-product-information_en.pdf>
