# Supplementary material for: Whole genome case-control study of central nervous system toxicity due to antimicrobial drugs
Source: PLoS One. 2024 Feb 29;19(2):e0299075. doi: 10.1371/journal.pone.0299075 (PMC10903854; doi:10.1371/journal.pone.0299075)
Supplement: S2 Table — (DOCX) [file pone.0299075.s008.docx]

**Table S2:** The 25 common variants with smallest p-value, all variants have a allele frequency larger then 0.123.

| **CHROM** | **POS** | **REF** | **ALT** | **OR** | **LOG(OR)_SE** | **P** |
| --- | --- | --- | --- | --- | --- | --- |
| 7 | 7398921 | CAA | CA | 4.25534 | 0.32185 | 6.81053E-06 |
| 7 | 24296239 | GAA | GA | 2.34425 | 0.189699 | 7.08408E-06 |
| 10 | 16796919 | C | A | 2.39203 | 0.194796 | 7.56253E-06 |
| 7 | 24280265 | CTTT | CTTTT | 2.38204 | 0.194032 | 7.70348E-06 |
| 10 | 16797033 | A | G | 2.36265 | 0.194668 | 1.00236E-05 |
| 12 | 16117301 | T | C | 0.375739 | 0.222097 | 1.04638E-05 |
| 19 | 44180595 | G | A | 2.31795 | 0.19225 | 1.22625E-05 |
| 8 | 32510642 | G | GT | 2.44362 | 0.204432 | 1.23934E-05 |
| 5 | 117231507 | A | T | 2.36487 | 0.197739 | 1.34389E-05 |
| 22 | 32656022 | A | G | 2.55047 | 0.215368 | 1.37798E-05 |
| 22 | 32656177 | C | T | 2.55047 | 0.215368 | 1.37798E-05 |
| 3 | 5943752 | A | G | 2.35021 | 0.196565 | 1.37892E-05 |
| 22 | 32647332 | T | C | 2.5503 | 0.215371 | 1.38026E-05 |
| 22 | 32648389 | A | T | 2.5503 | 0.215371 | 1.38026E-05 |
| 22 | 32649875 | A | C | 2.5503 | 0.215371 | 1.38026E-05 |
| 7 | 24293548 | C | T | 2.27857 | 0.190326 | 1.51124E-05 |
| 7 | 24293561 | G | A | 2.27857 | 0.190326 | 1.51124E-05 |
| 22 | 32650574 | A | G | 2.52065 | 0.213699 | 1.51664E-05 |
| 22 | 32649772 | CT | CTT | 2.52006 | 0.213717 | 1.52674E-05 |
| 22 | 32656788 | A | G | 2.50985 | 0.213793 | 1.67535E-05 |
| 22 | 32657104 | T | C | 2.50985 | 0.213793 | 1.67535E-05 |
| 22 | 32646746 | A | G | 2.50969 | 0.213796 | 1.67809E-05 |
| 22 | 32646756 | A | G | 2.50969 | 0.213796 | 1.67809E-05 |
| 2 | 213209342 | G | GT | 8.80863 | 0.505908 | 1.70302E-05 |
| 1 | 5068210 | A | G | 2.26498 | 0.190279 | 1.73386E-05 |
