# Supplementary material for: Whole genome case-control study of central nervous system toxicity due to antimicrobial drugs
Source: PLoS One. 2024 Feb 29;19(2):e0299075. doi: 10.1371/journal.pone.0299075 (PMC10903854; doi:10.1371/journal.pone.0299075)
Supplement: S3 Table — (DOCX) [file pone.0299075.s009.docx]

**Table S3:** top 10 genes in SKATO test

| **Gene** | **p-value** | **Variants included** | **q-value** |
| --- | --- | --- | --- |
| RETSAT | 8.42711806448015E-07 | 26 | 0.012906356933434 |
| LCP1 | 1.34092020087628E-06 | 18 | 0.012906356933434 |
| SFMBT2 | 5.48767734098732E-06 | 63 | 0.035212596271335 |
| SYNGR4 | 1.70939954979854E-05 | 8 | 0.082264853334055 |
| SESN2 | 0.000140009325528 | 19 | 0.450429870875879 |
| COX6C | 0.000140393725987 | 19 | 0.450429870875879 |
| POLR2G | 0.000219984196236 | 4 | 0.570173925155871 |
| NAA38 | 0.000236955397467 | 10 | 0.570173925155871 |
| LINC01124 | 0.000383959587987 | 5 | 0.821246896526762 |
| CD36 | 0.000502073569685 | 50 | 0.889143181609367 |
