# Supplementary material for: Whole genome case-control study of central nervous system toxicity due to antimicrobial drugs
Source: PLoS One. 2024 Feb 29;19(2):e0299075. doi: 10.1371/journal.pone.0299075 (PMC10903854; doi:10.1371/journal.pone.0299075)
Supplement: S4 Table — (DOCX) [file pone.0299075.s010.docx]

**Table S4:** Top variants present in genes significant in SKAT-O test

| **CHROM_POS:REF:ALT** | **gene** | **snp138** | **Control**  **Freq** | **Case**  **Freq** | **P** | **OR** | **LOG(OR SE)** |
| --- | --- | --- | --- | --- | --- | --- | --- |
| 13_46718722:T:C | LCP1 | rs6561297 | 0.0318127 | 0.121212 | 1.51046E-06 | 4.59291 | 0.316957 |
| 13_46722443:A:C | LCP1 | rs10492451 | 0.0312125 | 0.121212 | 1.51046E-06 | 4.59291 | 0.316957 |
| 2_85569952:A:G | RETSAT | rs60340620 | 0.027611 | 0.106061 | 2.10301E-05 | 3.95486 | 0.323238 |
| 2_85577310:C:T | RETSAT | rs139043592 | 0.00780312 | 0.0530303 | 4.27161E-05 | 7.36404 | 0.487897 |
| 2_85569925:G:A | RETSAT | rs145057327 | 0.0258103 | 0.0984848 | 4.81352E-05 | 3.85004 | 0.331673 |
| 2_85569942:G:A | RETSAT | rs572423656 | 0.0258103 | 0.0984848 | 4.81352E-05 | 3.85004 | 0.331673 |
| 2_85569944:A:T | RETSAT | rs192199548 | 0.0258103 | 0.0984848 | 4.81352E-05 | 3.85004 | 0.331673 |
| 2_85569947:A:T | RETSAT | rs183409558 | 0.0258103 | 0.0984848 | 4.81352E-05 | 3.85004 | 0.331673 |
| 2_85570442:C:T | RETSAT | rs143283662 | 0.0090036 | 0.0530303 | 0.000104488 | 6.37087 | 0.477261 |
| 2_85570146:G:A | RETSAT | rs4832167 | 0.0558223 | 0.128788 | 0.000745033 | 2.77647 | 0.302801 |
| 10_7244421:G:A | SFMBT2 | rs1830807 | 0.0210084 | 0.0681818 | 0.00117922 | 3.65188 | 0.399291 |
| 10_7239705:G:A | SFMBT2 | rs79856959 | 0.0348139 | 0.0909091 | 0.00293077 | 2.67872 | 0.331217 |
| 10_7201809:G:A | SFMBT2 | rs2692761 | 0.00960384 | 0.0378788 | 0.00776121 | 4.09539 | 0.529568 |
| 10_7214447:C:T | SFMBT2 | rs41302976 | 0.0756303 | 0.143939 | 0.00820968 | 2.00945 | 0.264009 |
| 10_7412243:G:A | SFMBT2 | rs142871492 | 0.00180072 | 0.0151515 | 0.0174483 | 9.0996 | 0.928951 |
| 10_7202931:C:T | SFMBT2 | rs2692759 | 0.0726291 | 0.128788 | 0.0212708 | 1.93366 | 0.286311 |
| 10_7262560:G:A | SFMBT2 | rs141321133 | 0.00540216 | 0.0227273 | 0.0325037 | 4.27539 | 0.679497 |
| 10_7205717:C:G | SFMBT2 | rs2692756 | 0.0762305 | 0.128788 | 0.0353673 | 1.81379 | 0.282975 |
| 2_85570273:G:A | RETSAT | rs7600799 | 0.0714286 | 0.121212 | 0.0378935 | 1.8091 | 0.285564 |
| 10_7201601:T:C | SFMBT2 | rs17422550 | 0.112845 | 0.174242 | 0.0446891 | 1.59993 | 0.234095 |
| 10_7200632:G:C | SFMBT2 | rs41289077 | 0.0030012 | 0.0151515 | 0.0491404 | 5.32129 | 0.84972 |
| 10_7200890:A:T | SFMBT2 | rs17142544 | 0.00360144 | 0.0151515 | 0.0491404 | 5.32129 | 0.84972 |
| 10_7203638:C:G | SFMBT2 | rs142046083 | 0.0030012 | 0.0151515 | 0.0491404 | 5.32129 | 0.84972 |
| 10_7204407:G:A | SFMBT2 | rs41290251 | 0.0030012 | 0.0151515 | 0.0491404 | 5.32129 | 0.84972 |
| 10_7204992:G:GA | SFMBT2 | . | 0.0030012 | 0.0151515 | 0.0491404 | 5.32129 | 0.84972 |
| 10_7205430:G:A | SFMBT2 | rs41290253 | 0.0030012 | 0.0151515 | 0.0491404 | 5.32129 | 0.84972 |
| 10_7214182:G:A | SFMBT2 | rs61836740 | 0.0030012 | 0.0151515 | 0.0491404 | 5.32129 | 0.84972 |
| 13_46704901:C:T | LCP1 | . | 0.00060024 | 0.00757576 | 0.0740474 | 12.6382 | 1.42008 |
| 2_85569379:C:T | RETSAT | rs15413 | 0.0714286 | 0.113636 | 0.083491 | 1.6488 | 0.288915 |
| 13_46701876:G:A | LCP1 | rs185641235 | 0.00420168 | 0.0151515 | 0.11056 | 3.65574 | 0.812379 |
| 10_7204830:C:T | SFMBT2 | rs143217875 | 0.00420168 | 0.0151515 | 0.113932 | 3.61157 | 0.812357 |
| 10_7214418:C:A | SFMBT2 | rs373469070 | 0.00120048 | 0.00757576 | 0.138275 | 6.20843 | 1.23185 |
| 2_85570463:C:T | RETSAT | rs142803140 | 0.00480192 | 0.0151515 | 0.15269 | 3.14587 | 0.801409 |
| 10_7204129:C:T | SFMBT2 | rs41290249 | 0.00540216 | 0.0151515 | 0.193349 | 2.8036 | 0.792556 |
| 10_7205085:CAACAAACA:CAACAAACAAACA | SFMBT2 | . | 0.0060024 | 0.0151515 | 0.193743 | 2.80118 | 0.79259 |
| 10_7242441:G:A | SFMBT2 | rs41306403 | 0.015006 | 0.030303 | 0.195292 | 2.05085 | 0.554601 |
| 2_85570024:A:G | RETSAT | rs57986502 | 0.0996399 | 0.128788 | 0.281497 | 1.37382 | 0.2949 |
| 10_7218202:C:T | SFMBT2 | rs140501686 | 0.0306122 | 0.0151515 | 0.302562 | 0.469903 | 0.732551 |
| 10_7230572:T:C | SFMBT2 | rs201763292 | 0.00240096 | 0.00757576 | 0.30812 | 3.14922 | 1.12557 |
| 2_85571285:C:T | RETSAT | rs41289947 | 0.00720288 | 0.0151515 | 0.343392 | 2.08408 | 0.775028 |
