# Supplementary material for: Whole genome case-control study of central nervous system toxicity due to antimicrobial drugs
Source: PLoS One. 2024 Feb 29;19(2):e0299075. doi: 10.1371/journal.pone.0299075 (PMC10903854; doi:10.1371/journal.pone.0299075)
Supplement: Attachment — Submitted filename: Response to Reviewers.docx [file pone.0299075.s011.docx]

PONE-D-23-09336

Whole genome case-control study of central nervous system (CNS) toxicity due to antimicrobial drugs

Journal Requirements:

When submitting your revision, we need you to address these additional requirements.

1. Please ensure that your manuscript meets PLOS ONE's style requirements, including those for file naming. The PLOS ONE style templates can be found at

<https://journals.plos.org/plosone/s/file?id=wjVg/PLOSOne_formatting_sample_main_body.pdf> and

<https://journals.plos.org/plosone/s/file?id=ba62/PLOSOne_formatting_sample_title_authors_affiliations.pdf>

*Reply: We have now formatted the manuscript according to PLOS ONE's style requirements.*

2. Thank you for stating the following financial disclosure:

"This study was granted by the Science for Life Laboratory’s Swedish Genomes Program 2017 that was supported by the Knut and Alice Wallenberg Foundation (application ID NP:00085). Furthermore, we received grants from the Swedish Research Council (Medicine 521-2011-2440, 521-2014-3370 and 2018-03307), and Clinical Research Support (Avtal om Läkarutbildning och Forskning, ALF) at Uppsala University. All grants mentioned were granted to MW and PH."

Please state what role the funders took in the study.  If the funders had no role, please state: "The funders had no role in study design, data collection and analysis, decision to publish, or preparation of the manuscript."

If this statement is not correct you must amend it as needed.

Please include this amended Role of Funder statement in your cover letter; we will change the online submission form on your behalf.

*Reply: The role of the funder has been added to the financial disclosure on page 23 (in the version with tracked changes) and to the cover letter. "The funders had no role in study design, data collection and analysis, decision to publish, or preparation of the manuscript."*

3. Thank you for stating the following in the Competing Interests section:

"The authors declare no conflict of interest. No funder took part in the study design, recruitment, analysis, interpretation of data, writing of the report or in the decision to submit the paper for publication. There are no other conflicts of interest."

Please confirm that this does not alter your adherence to all PLOS ONE policies on sharing data and materials, by including the following statement: "This does not alter our adherence to  PLOS ONE policies on sharing data and materials.” (as detailed online in our guide for authors <http://journals.plos.org/plosone/s/competing-interests>).  If there are restrictions on sharing of data and/or materials, please state these. Please note that we cannot proceed with consideration of your article until this information has been declared.

Please include your updated Competing Interests statement in your cover letter; we will change the online submission form on your behalf.

*Reply: Conditions for data sharing have been moved from the Data Availability Statement to the Competing Interests statement on page 23, and are also written in the cover letter. “Genetic data will be available at* *the official Federated European Genome-phenome Archive (FEGA) at NBIS/Elixir (https://fega.nbis.se/). Data access can be requested if the recipient adheres to our ethics approval, and the request is in accordance with FAIR data management and the General Data Protection Regulation (GDPR) 2016/679.”*

4. Please include your full ethics statement in the ‘Methods’ section of your manuscript file. In your statement, please include the full name of the IRB or ethics committee who approved or waived your study, as well as whether or not you obtained informed written or verbal consent. If consent was waived for your study, please include this information in your statement as well.

*Reply: The ethics statement has been moved from the Informed Consent statement at the end of the manuscript to the Methods section. See 2.1 on page 5 “The study was conducted according to the guidelines of the Declaration of Helsinki, and the study was approved by the regional ethical review board in Uppsala, Sweden (2010/231 Uppsala). Written informed consent was obtained from all participants in the study.”*

5. In your Data Availability statement, you have not specified where the minimal data set underlying the results described in your manuscript can be found. PLOS defines a study's minimal data set as the underlying data used to reach the conclusions drawn in the manuscript and any additional data required to replicate the reported study findings in their entirety. All PLOS journals require that the minimal data set be made fully available. For more information about our data policy, please see <http://journals.plos.org/plosone/s/data-availability>.

Upon re-submitting your revised manuscript, please upload your study’s minimal underlying data set as either Supporting Information files or to a stable, public repository and include the relevant URLs, DOIs, or accession numbers within your revised cover letter. For a list of acceptable repositories, please see <http://journals.plos.org/plosone/s/data-availability#loc-recommended-repositories>. Any potentially identifying patient information must be fully anonymized.

Important: If there are ethical or legal restrictions to sharing your data publicly, please explain these restrictions in detail. Please see our guidelines for more information on what we consider unacceptable restrictions to publicly sharing data: <http://journals.plos.org/plosone/s/data-availability#loc-unacceptable-data-access-restrictions>. Note that it is not acceptable for the authors to be the sole named individuals responsible for ensuring data access.

*Reply: Data will be made available as described above. As the minimal data consists of sensitive data the data cannot be shared without recipient adhering to our ethics approval, and GDPR. For this, an individual data management access approval contract needs to be signed.*

We will update your Data Availability statement to reflect the information you provide in your cover letter.

[Note: HTML markup is below. Please do not edit.]

Reviewers' comments:

Reviewer's Responses to Questions

**Comments to the Author**

1. Is the manuscript technically sound, and do the data support the conclusions?

The manuscript must describe a technically sound piece of scientific research with data that supports the conclusions. Experiments must have been conducted rigorously, with appropriate controls, replication, and sample sizes. The conclusions must be drawn appropriately based on the data presented.

Reviewer #1: Yes

Reviewer #2: Partly

2. Has the statistical analysis been performed appropriately and rigorously?

Reviewer #1: Yes

Reviewer #2: No

3. Have the authors made all data underlying the findings in their manuscript fully available?

The [PLOS Data policy](http://www.plosone.org/static/policies.action#sharing) requires authors to make all data underlying the findings described in their manuscript fully available without restriction, with rare exception (please refer to the Data Availability Statement in the manuscript PDF file). The data should be provided as part of the manuscript or its supporting information, or deposited to a public repository. For example, in addition to summary statistics, the data points behind means, medians and variance measures should be available. If there are restrictions on publicly sharing data—e.g. participant privacy or use of data from a third party—those must be specified.

Reviewer #1: Yes

Reviewer #2: Yes

4. Is the manuscript presented in an intelligible fashion and written in standard English?

PLOS ONE does not copyedit accepted manuscripts, so the language in submitted articles must be clear, correct, and unambiguous. Any typographical or grammatical errors should be corrected at revision, so please note any specific errors here.

Reviewer #1: Yes

Reviewer #2: Yes

5. Review Comments to the Author

Please use the space provided to explain your answers to the questions above. You may also include additional comments for the author, including concerns about dual publication, research ethics, or publication ethics. (Please upload your review as an attachment if it exceeds 20,000 characters)

**Reviewer #1**: General comments:
Thank you for giving me the opportunity to review this manuscript. This paper is interesting because data that are important towards understanding the genetic basis of antimicrobial-induced CNS toxicity are still lacking.

Specific comments:
Good manuscript writing. Authors may refer to article “STrengthening the REporting of Genetic Association Studies (STREGA)— An Extension of the STROBE Statement” to improve the writing.

*Reply: We have completed the STROBE form.*

However,

Title and Abstract:
Please indicate the study’s design with a commonly used term in the title or the abstract.

*Reply: Thank you for pointing this out. The word “case-control” has been added to the title.*

Materials and Methods:
Study design: Please present key elements of study design early in the paper.

*Reply: The study design is presented in the title and elaborated on in the abstract. Aims are reiterated in the last paragraph of the introduction (pages 3-4) and we have added a starting paragraph to the materials and methods (page 5) describing key elements of the study design.*

Participants: Give the eligibility criteria (e.g. no exclusion criteria) and methods of selection of participants (sampling methods)

*Reply: Patients with a reported suspected CNS-toxic ADR were eligible, which has been added to page 5. Patients needed to be at least 18 years of age at the time of recruitment. Each patient was adjudicated and any patient with a suspected peripheral neurotoxicity was excluded (3.1 patient characteristics, page 12).*

Variables: Clearly define all outcomes, exposures, predictors, potential confounders, and effect modifiers

*Reply:*

- *Exposure:*
  - *Treatment with antimicrobial drugs*
- *Main Outcome and Measures:*
  - *Presence of a CNS-toxic adverse drug reaction*
  - *Correlation of genetic variation between cases and controls*
- *Effect modifiers:*
  - *As the controls are not matched for drug treatment, we cannot exclude the possibility of some being at risk for CNS-toxic reactions. If there is a causal genetic predisposition this possibility could underestimate the observed correlations. This means that the correlations observed can be viewed as conservative.*
- *Potential confounders:*
  - *With unmatched controls, the possibility of cofounding by indication exists. However, as the indications of cases are different types of infections or the preventive treatment for malaria, we do not believe that there is a genetic predisposition for these indications.*

*An overview of all drugs and CNS-toxic reactions among cases can be found in* ***Table 2.***

Data sources measurement:
• Describe laboratory methods, including storage of DNA.

*Reply: Laboratory methods are described on page 7. DNA storage has been added to page 6.*

• State the laboratory/centre where genotyping was done.

*Reply: The laboratory is described on page 6.*

• Describe comparability of laboratory methods if there is more than one group.

*Reply: Only one laboratory was used.*

• Specify whether genotypes were assigned using all of the data from the study simultaneously or in smaller batches.

*Reply: The full cohort wis sequenced in four batches. A clearer outline of these batches has been written under 2.4 on page 6.*

Bias: Describe any efforts to address potential sources of bias

*Reply: Samples with sequencing results of insufficient quality were re-sequenced. The cases were spread randomly throughout the batches. Individuals that were part of a study with strong known association to HLA-type were excluded (2.4, page 6).*

Study size: Explain how the study size was arrived at.

*Reply: Attempts were made to include all available cases that fulfilled the criteria in Sweden 1990-2017. Recruitment was performed 2010-2017.*

Statistical methods:
• Explain how missing data were addressed.

*Reply:*

*Missing data:*

- *Variant missingness: any variants with over 5% missing genotyping were excluded (2.7, page 8)*
- *Patient: NGI-U re-sequenced any failed samples (2.4, page 6)*
- *Parent’s country of birth was only used to validate PCA results. Outliers in genomic PCA were excluded (2.4, page 6). The current PCA plot (Fig S1) only shows the included cases and controls.*

• State whether Hardy-Weinberg equilibrium was considered and, if so, how.

*Reply: Hardy-Weinberg equilibrium:*

- *Any variant below the HWE threshold p < 10^-8 among controls was excluded (2.7, page 8)*

• Describe any methods used to address and correct for relatedness among subjects

*Reply: Methods used to address and correct for relatedness:*

- *The first 4 principal components were used as covariates in all tests performed.*

Notes: I am not able to access the supplementary files (tables/figures)

**Reviewer #2**: This paper explored whether any genetic variation across the whole genome was associated with risk of CNS toxicity due to antimicrobial drugs. Whole genome sequencing of 66 cases and 833 controls was performed. According to SKAT-O results, authors conclude CNS toxicity due to antimicrobial drugs was associated with uncommon variants in LCP1, RETSAT and SFMBT2.
There are some problems, which must be solved before the paper is considered for publication.

First, to apply rigorous quality control (QC) procedures. For example, in PCA figure (S1) some of the Swedegene dots are decentralized and some are even closer to the CHB and JPT populations, which imply the data need to further analyze. Sequencing quality or depth difference of case/control group in the positive regions should also be examined.

*Reply: Thank you for your insightful comments. We took several measures that address your concerns relating to QC procedures in terms of sequence quality and sequencing depth deviation. We used multiple QC statistics and methods to look for inconsistencies between samples, regions and batches. NGI-U, who did the sequencing and initial pre-processing, delivered the data as recalibrated bam-files. NGI-U only released data that met their stringent quality criteria. In cases where samples did not meet these standards, they opted to re-sequence rather than provide subpar data. Due to this, there were no highly divergent outliers in terms of depth, coverage or sequencing quality. This is explained under Materials and Methods on pages 6-8.*

*Thank you for pointing out the outlier in the population stratification plot (supplementary fig 1). In the previously submitted figure, it was not possible to discern whether the outlier was a case or a control. For enhanced clarity, we redrew the figure, and cases are now represented as squares and controls as circles. We agree that the single control that is closer to the CHB and JPT population could have been excluded. However, it is one of 833 controls, and its possible impact on the results is therefore negligible. We believe that this holds particularly true when examining the variants identified as driving the SKAT-O associations. Notably, these variants exhibit a significantly elevated variant frequency. It is essential to underscore that the maximum impact on variant frequency among controls is capped at +/- 1.2*10 ^-3. An important note here is that it could have been problematic if the outlier had been one of the cases, due to the higher influence this would have among the relatively few cases, where the largest effect would have been 1.5 * 10^-2.*

Second, considering the small sample size (especially case group), all negative results of association analysis need to be reduced in length (due to limited power).

*Reply: The supplemental lists of SKAT-O and common variant analysis have been reduced by half in length (from 20 to 10, and from 50 to 25)*

Third, positive results in LCP1, RETSAT and SFMBT2 gene mainly relies on SKAT-O analysis. The conclusion is not solid unless more evidence is added (such as replication in another population, population stratifications/subgroups analysis, gene set analysis or gene function evidence).

*Reply:*

- *Replication in another population*
  - *This is the long-term goal of this study, the problem with these ADRs is that they are rare, CNS-toxicity is heterogeneous, there is a multitude of antimicrobial drugs and in addition these ADRs are woefully underreported in healthcare. In order to expedite future research, any possible association found by our or another group needs to be communicated. Unfortunately, we do not know of any other material or studies that we could use for this today.*
- *Subgroups analysis*
  - *As the number of cases are small, we believe that increasing the complexity and decreasing the number of cases by performing a subgroup analysis would not be fruitful. While we would have liked to do this, we are of the opinion that a larger number of cases would have been required.*
- *Gene set analysis*
  - *Due to the heterogeneity of the question, we believe that it would be challenging to derive meaningful conclusions from enrichment tests. Analyzing previous studies that include findings related to LCP1, RETSAT, and SFMBT2, it appears that any direct relevance to our question is, perhaps, marginal. Consequently, combining these factors would likely result in an even more intricate outcome, making interpretation more difficult, in our opinion.*
- *Gene function evidence*
  - *The associated genes or their proteins, have previously been linked to symptoms that could be due to neurotoxicity, as discussed on pages 20-21.*

Some other minor issues:
Line 277-278, p value and VF of two variants are exactly the same (inconsistency with description elsewhere in the text).

*Reply: Thank you for bringing the discrepancy between the abstract and the results to our attention. The figures presented in the results and table S4 were correct. The two SNPs are in almost complete LD, and both SNPs have a variant frequency of 3% among cases. The abstract has now been updated (page 2).*

Uncommon variants frequency (define as VF < 0.123 in the paper) is need for clearer rationale and explanation.

*Reply: We now describe how the variant frequency (VF) threshold was calculated using the Hardy-Weinberg principle with an expected value of one homozygote individual among cases, on page 9. “66 cases equate a threshold of 1/√66 = 0.123.”*

6. PLOS authors have the option to publish the peer review history of their article ([what does this mean?](https://journals.plos.org/plosone/s/editorial-and-peer-review-process#loc-peer-review-history)). If published, this will include your full peer review and any attached files.
If you choose “no”, your identity will remain anonymous but your review may still be made public.

**Do you want your identity to be public for this peer review?** For information about this choice, including consent withdrawal, please see our [Privacy Policy](https://www.plos.org/privacy-policy).

Reviewer #1: **Yes:** Zalina Zahari

Reviewer #2: **Yes:** Jian Guo
